# Pituitary Cachexia Treated with Corticotrophic Hormone

**Published:** 1946

**Authors:** R. E. Hemphill

**Affiliations:** Medical Superintendent, Bristol Mental Hospital; Lecturer in Psychiatry, University of Bristol


### PITUITARY CACHEXIA TREATED WITH CORTICOTROPIC HORMONE

BY

R. E. HEMPHILL, M.A., M.D., D.P.M.

*Medical Superintendent, Bristol Mental Hospital;*
*Lecturer in Psychiatry, University of Bristol.*

EXTREME emaciation accompanied by a melancholic psychosis is sometimes seen in women at or, more frequently, after the menopause. A number of such cases have been investigated and treated at the Bristol Mental Hospital, so that it is possible to describe this form of Involutional Melancholia as a clinical entity (Hemphill and Reiss, 1940 and 1942).

Physically there is gross emaciation with loss of muscle strength; the skin is inelastic and very dry, having a dessicated appearance and texture; there is a complete absence of sweating; the breasts and external genitalia are atrophied, axillary and pubic hair falls out while hair of the head and eyebrows is brittle and lustreless; the thyroid is usually small and menses have ceased. In some cases a vaginal smear shows cell changes of atrophy. Subjectively patients complain of weakness, sensation of cold and loss of appetite. They constantly express the delusion that there is some abnormality of the intestinal tract, so that food does not pass through it, but if swallowed it appears to be passed into some other part of the body, such as the bladder or down the legs. The patients look sallow, weak and ill. They move little, thus conserving energy, so that their behaviour is in marked contrast to the restless, tense activity and strength of anorexia nervosa.

There are no typical biochemical or blood changes, but the output of gonadotropic, thyrotropic and corticotropic hormones has been nil or very low in every case in which these estimations have been made. The output of 17-keto-steroids per 24 hours is very low or nil.

The whole picture closely resembles that of Simmond's disease, and the condition can be fairly attributed to a reduced output of anterior pituitary hormones at or after the menopause without the structural change in the pituitary of Simmond's disease.

I have treated a number of these patients with corticotropic hormone, on the assumption that the weakness, changes in the skin, loss of appetite and the delusions of disturbances in the alimentary tract were in the main due to adreno-cortical insufficiency. This assumption is supported by the low output of 17-keto-steroids which in the normal female are entirely derived from the adrenal cortex.

The results in well defined cases have been encouraging and an account of one extreme case, with photographs, has been published (Hemphill and Reiss, 1944). Through the courtesy and co-operation of Dr. H. H. Carleton I have had the opportunity of treating another very similar case in the Bristol Royal Infirmary, an account of which follows.

D.P.F. Age 47. Married. Was admitted to Bristol Royal Infirmary January 4th, 1946, with the following history: In March, 1945, she had a severe attack of diarrhoea and vomiting, during which time she fainted frequently. Two months later she developed the idea that her food went into the back of her neck or down the left side into her bladder. From this date she lost her appetite and ate very little. She said that at times she felt hungry, but food was not going into her stomach, and so it was no good eating. Menses had ceased abruptly 18 months before.

Her previous history showed that she had suffered from indigestion and had had nervous breakdowns, none of which were sufficient to have her admitted to hospital. She had recently been nursing a member of the family who suffered from cancer of the breast. Family History: Father and mother dead, cause unknown. Sister well. No children.

On admission she looked ill, seemed very depressed and moved very little in bed. Skin was loose and dry. There was no obvious disorder of any system. There was marked loss of weight, little subcutaneous fat, hair was brittle, and was falling out of head and eye-brows; it was very scanty in the axillae and on pubis. Breasts and external genitalia were extremely atrophied. Mucous membrane and tongue were normal, and did not indicate a vitamin deficiency. It was suspected that there might be a carcinoma of the stomach. Full investigations revealed nothing abnormal in intestinal tract, heart or lungs. Blood examination: R.B.C. 4,660,000. Hb. 90 per cent. C.I. 0.96. W.B.C. 8,600. Fractional Test-Meal was within normal limits. Weight was 4 stone 13 lb. Output of 17-keto-steroids was 4.0 mg. per twenty-four hours.

On January 28th corticotropin, 10 sudanophobic units three times a day and vitamin B supplement were administered. Corticotropin was given for one week, there was one week's rest, and then a further course of two weeks. Within the first week there was immediate improvement. The patient began to eat and to lose the delusion that food passed down the back of her neck and into the left side of her bladder. She maintained that she was hungry, and enjoyed her food. Her skin lost the inelastic appearance, became flushed and sweated. In one month (February 28th, 1946), her condition was greatly improved, her general appearance and skin was normal. There was evidence of eye-brows beginning to grow, and the hair elsewhere seemed to be getting thicker, definitely altered in texture, becoming more supple and shiny. Her weight at this time was 6 stone 1½ lb.

The patient was discharged from hospital March 5th, 1946, that is just five weeks after the commencement of treatment. She has remained well ever since, has shown no tendency to relapse and her weight, two months later, was 6 stone 12 lb.

In the case described above, all the criteria were satisfied so that a definite diagnosis could be made. The emaciation, condition of skin and hair, strength of muscles, loss of strength, and the mental picture corresponded. There was an immediate improvement with corticotropic hormone treatment.

The action of this hormone is to stimulate the adrenal cortex into activity. One must assume that the hypo-pituitarism in this case is not extreme, and as improvement and recovery follows treatment with corticotropic hormone it seems likely that the normal regulation of other glands, such as the thyroid, by the pituitary becomes automatically established when once one part of the physiological change has been corrected.

It is impossible to say how common these cases are. It may be that this condition only occurs in subjects with an abnormal nervous constitution, and it may be that starvation is partly responsible. Be that as it may, experience in the mental hospital is that no amount of feeding by itself is effective and that the response to corticotropic hormone is immediate and may be dramatic. The consistent delusion that the alimentary tract is stopped up or that food passes into some abnormal channel is due to psychotic misinterpretation of unusual sensations in the alimentary tract, which presumably would not be met in an individual of normal mental make-up. As soon as the condition of the gut and the appetite are restored the delusion disappears and the patient seems to forget it afterwards. I have not observed clear-cut cases in males, probably because there is no endocrine disturbance that closely corresponds with the menopause in the female. In all cases studied so far the menopause has been sudden and not marked by the usual menopausal symptoms, as flushing, sweating, tremors. This may be a significant point. Treatment does not need to be prolonged, and it seems that once the endocrine mechanism has been got to work normally corticotropic hormone need no longer be given, as it is then produced by normal action of the pituitary.

I am much indebted to Dr. H. H. Carleton for permission to examine and treat this case, and to publish this paper.

REFERENCES.

Hemphill, R. E., and Reiss, M., 1940, *Journ. Ment. Sci.*, 86, 1065.
Hemphill, R. E., and Reiss, M., 1942, *Journ. Ment. Sci.*, 88, 1.
Hemphill, R. E., and Reiss, M., 1944, *Brit. Med. Journ.*, 2, 211.

